# Depressive disorders among patients with gastric cancer in Taiwan: a nationwide population-based study

**DOI:** 10.1186/s12888-018-1859-8

**Published:** 2018-09-03

**Authors:** Li-Yu Hu, Chia-Jen Liu, Chiu-Mei Yeh, Ti Lu, Yu-Wen Hu, Tzeng-Ji Chen, Pan-Ming Chen, Shyh-Chyang Lee, Cheng-Ho Chang

**Affiliations:** 1 Department of Psychiatry, Kaohsiung Veterans General Hospital, No.386, Dajhong 1st Rd., Zuoying Dist., Kaohsiung City, 813 Taiwan; 2 Department of Psychiatry, Taipei Veterans General Hospital, Taipei, Taiwan; 3 Center for Geriatrics and Gerontology, Kaohsiung Veterans General Hospital, Kaohsiung, Taiwan; 4 School of Medicine, National Yang-Ming University, Taipei, Taiwan; 5 Division of Hematology and Oncology, Department of Medicine Taipei Veterans General Hospital, Taipei, Taiwan; 6 Department of Family Medicine, Taipei Veterans General Hospital, Taipei, Taiwan; 7 Cancer Center, Taipei Veterans General Hospital, Taipei, Taiwan; 8 Department of Psychiatry, Yuanshan & Su-ao Branch, Taipei Veterans General Hospital, Yilan, Taiwan; 9 Department of Information Management, Chia Nan University of Pharmacy & Science, Tainan, Taiwan; 10 Department of Orthopedics, Chiayi Branch, Taichung Veterans General Hospital, Chiayi, Taiwan

**Keywords:** Depression, Epidemiology, Gastric neoplasms, Risk factor

## Abstract

**Background:**

In cancer patients, depressive disorder comorbidity is associated with greater suicide risk and poorer treatment outcomes, quality of life, and adherence to treatment. The aim of the study was to evaluate the incidence of newly-diagnosed depressive disorders after a gastric cancer diagnosis compared with a matched cohort using the National Health Insurance Research Database in Taiwan.

**Methods:**

We conducted a retrospective cohort study of 57,506 patients (28,753 patients with gastric cancer and 28,753 matched patients) selected from the National Health Insurance Research Database. Patients were observed for a maximum of 12 years to determine the incidence of newly-diagnosed depressive disorders. Also, a Cox regression analysis which included death as an independent censor was performed to identify the potentially predictive variables for developing subsequent depressive disorders following a cancer diagnosis among the patients suffering from gastric cancer.

**Results:**

The cumulative incidence of depressive disorders in the gastric cancer patients was significantly higher compared to those in the matched cohort (*p* < .001). The adjusted hazard ratio was 1.54 (95% confidence interval, CI = 1.39–1.70, *P* < .001) in the gastric cancer cohort compared with the matched cohort. Independent predictive variables for developing subsequent depressive disorders among the patients with gastric cancer included female sex and hypertension.

**Conclusions:**

In the study, higher incidence of new-onset depression, being defined by the records of the diagnostic codes combining antidepressants use in a nationwide database, was noted in the gastric cancer patients compared with the matched cohort. In addition, female sex and comorbid hypertension may be predictive variables for the subsequent depression among the patients with gastric cancer. Further clinical prospective studies were necessary to confirm these findings.

## Background

Although the incidence of gastric cancer has declined over the last few decades in many countries [1–4], it is still one of the most common cancers in the world. In 2012, an estimated 951,600 newly diagnosed gastric cancer patients and 723,100 deaths occurred worldwide [5]. Gastric cancer rates are generally about twice as high in men as they are in women [6]. Most important, the incidence rates of gastric cancer vary widely across countries. Evidence has showed that incidence rates are highest in Eastern Asia, Central and Eastern Europe, and South America. In addition, more than half of newly diagnosed gastric cancer cases occurring in Eastern Asia [5, 7]. For example, in 2012, there were 3795 Taiwanese being diagnosed as gastric cancer and the gastric cancer was the fifth-most frequently diagnosed cancer with a age-standardized incidence rate of 15.03 per 100,000 people [8]. Moreover, gastric cancer has also a high mortality; for instance, in Taiwan, gastric cancer ranks as the sixth-highest cause of cancer-related deaths in 2012 with a standardized mortality rate of 6.4 per 100,000 people [7].

Cancer is a risk factor for developing emotional disturbances, especially depression [9]. Evidence has revealed that depressive disorders are associated with reduced quality of life and poorer outcomes in patients with cancer [10]. Several studies found an increased risk of suicide among patients with cancer compared with the general population [11–13]. Additionally, depression has also been associated with poor survival among gastric cancer patients in China [14].

Depression is the most prevalent psychiatric disorder among the various types of cancer patients. Massieet et al. reviewed previous studies regarding the prevalence of depression in patients with cancer and found that the reported prevalence (major depressive disorder: 0–38%; depression spectrum syndromes: 0–58%) varied significantly because of different conceptualizations of depression, criteria used to define depression, methodological approaches to the measurement of depression, and study populations [15]. Depression has been studied in patients with cancer using a range of assessment methods, such as self-report rating scales, brief screening instruments, and structured clinical interviews. The methods commonly used are the Hospital Anxiety and Depression Scale, Beck Depression Inventory, Hamilton Rating Scale for Depression questionnaire, and the Diagnostic and Statistical Manual of Mental Disorders criteria, published by the American Psychiatric Association. In general, the more narrowly the term is defined, the lower the reported prevalence of depression [15]. Bergquist et al. found that the rate of depression among those with esophagus or gastroesophageal junction cancer ranged from 27 to 44% [16]. Among these studies, diagnoses were usually not clinically defined and there have been few epidemiological studies specifically of depressive disorders among gastric cancer patients.

To reduce the evidence-to-practice gap and overcome the abovementioned shortage of previous studies and based on the fact that higher incidence and mortality rates of gastric cancer in Taiwan by annual government report and epidemiological studies, we designed a nationwide population-based retrospective cohort study, using a different definition for depressive disorders, to investigate the clinical pictures among the patients with gastric cancer and try to explore the differences on the incidence of subsequent depressive disorders compared with the matched cohort using the Taiwanese National Health Insurance Research Database (NHIRD). Also, independent predictive variables for subsequent depressive disorders following a gastric cancer diagnosis were also analyzed.

## Methods

### Data sources

The Taiwan National Health Insurance (NHI) program, which the government initiated in 1995, provides comprehensive health care for all Taiwanese residents. Enrollment in this program is mandatory, and the proportion of the population insured reached 99% in 2006 [17]. The NHIRD contains information regarding clinical visits, including prescription details and diagnostic codes based on the International Classification of Diseases, Ninth Revision, Clinical Modification (ICD-9-CM). The NHIRD is managed by the National Health Research Institutes (NHRI) and confidentiality is maintained according to the directives of the NHI Bureau. This database also provides comprehensive use of and enrollment information for all patients with “catastrophic illnesses.” Catastrophic illnesses are a set of diseases, such as cancer, defined by the Taiwanese government; patients with catastrophic illness certificates are exempt from copayments under the NHI program. Application for catastrophic illness certificates requires sufficient medical records and an independent peer review process. Therefore, in our study, the diagnoses of gastric cancer were valid. Patients with gastric cancer and matched patients were identified from the NHI database. The Institutional Review Board of the Taipei Veterans General Hospital approved this study (2013-06-010 BC). Written consent from the study patients was not required because the NHI dataset consists of de-identified secondary data used for research purposes, and the Institutional Review Board of the Taipei Veterans General Hospital issued a formal written waiver regarding the need for consent.

### Study population

Using the discharge codes (151.X) (Malignant neoplasm of stomach) of the ICD-9-CM in the Registry of Catastrophic Illness, we identified 28,753 patients who were newly diagnosed with gastric cancer between January 1, 2000, and December 31, 2011. We excluded patients under 20 years of age and those who were diagnosed with depressive disorders prior to the cancer diagnosis. Depressive disorders were defined according to the relevant ICD-9-CM codes (depressive disorders: 296.2×–296.3×, 300.4, 311.x) [18]. In addition, we collected information on the antidepressant prescriptions that could be used to treat depressive disorders. In order to ensure the validity of definition for depressive disorders in the study, the development of depressive disorders was based on the ICD-9-CM codes and the prescription of antidepressants by at least one qualified psychiatrist for at least 30 days [18, 19]. However, the definition of depressive disorders in the study should not be misinterpreted as to look upon antidepressants use or any other biological interventions as the major role in addressing depression among the patients with gastric cancer. For each gastric cancer patient included in the final cohort, one age-, sex-, common-comorbidity-, and enrollment-date-matched patient who was not diagnosed with gastric cancer was randomly selected from the same database.

### Statistical analyses

The main dependent variable was the incidence of comorbid depressive disorders. The two cohorts were observed until the development of depressive disorders, death, withdrawal from the NHI system, or December 31, 2011. Each patient was followed for a maximum of 12 years. Incidence rates (per 1000 person-years) were analyzed. Formal comparisons between groups were performed using the chi-squared test for categorical variables and Mann-Whitney U test for continuous variables. Cumulative incidences of depressive disorders were calculated and stratified by gastric cancer and the matched cohort. The Kaplan-Meier method estimated the cumulative incidence and overall survival rate. A Cox proportional hazards model was used to identify risk factors for depressive disorders and patient death was included as an independent censor. Information about gastric cancer treatments, such as major surgery—including total gastrectomy, esophagogastrectomy, chemotherapy, and radiotherapy—and other control variables—such as age, sex, and comorbidities—were included in the model. Moreover, gastric cancer treatments were analyzed as a time-dependent covariate in order to avoid immortal time bias [20] and the Fine-Gray model was used for controlling the competing risk of death while calculating the incidence of newly-diagnosed depressive disorders. The data were extracted and computed using the Perl programming language (Version 5.12.2; Perl Foundation, Walnut, CA, USA). Microsoft SQL Server 2012 (Microsoft Corp., Redmond, WA, USA) was used for data linkage, processing, and sampling. SAS 9.2 software (SAS Institute Inc., Cary, NC, USA) or Stata statistical software (Version 11.0; Stata Corp, College Station, TX, USA) was used in the statistical analyses. A *p* value < .05 was considered statistically significant.

## Results

### Clinical characteristics of the study population

During the study period, 28,753 patients with gastric cancer were enrolled. The interquartile range for patient age was55–77 years (Median age = 69 years). The top three comorbidities were hypertension (49.7%), chronic obstructive pulmonary disease (COPD) (29.9%), and ischemic heart disease (29.7%). The demographic data and comorbidities of the patients with gastric cancer and the matched group are shown in Table 1. Age, sex, and all of the listed comorbid diseases were matched. The median follow-up period for the matched group was 4.75 years, which was significantly longer than the 1.23-year median follow-up period observed in the gastric cancer cohort (*P* < .001 according to the Mann-Whitney U test).

**Table 1:** Baseline patient characteristics of patients with and without gastric cancer

Demographic data	Patients with gastric cancer *n* = 28,753		Matched cohort *n* = 28,753		*P* value
	*n*	%	*n*	%	
Age (years) (interquartile range)	69 (55–77)		69 (55–77)		
≥65	16,889	58.7	16,889	58.7	1.000
< 65	11,864	41.3	11,864	41.3	.
Sex					
Male	19,464	67.7	19,464	67.7	1.000
Female	9289	32.3	9289	32.3	.
Comorbidities					
Diabetes mellitus	7895	27.5	7894	27.5	0.993
Hypertension	14,302	49.7	14,304	49.7	0.987
Heart failure	3712	12.9	3711	12.9	0.990
COPD	8586	29.9	8584	29.9	0.985
Chronic kidney disease	4123	14.3	4122	14.3	0.991
Cirrhosis	1469	5.1	1420	4.9	0.350
Autoimmune diseases	1721	6	1719	6	0.972
Cerebrovascular disease	5565	19.4	5567	19.4	0.983
Ischemic heart disease	8549	29.7	8550	29.7	0.993
Follow-up years (median)	1.23 (0.41–3.68)		4.75 (2.19–7.89)		< 0.001

*COPD* chronic obstructive pulmonary disease

### Incidence rates of depressive disorders

**Fig. 1:**
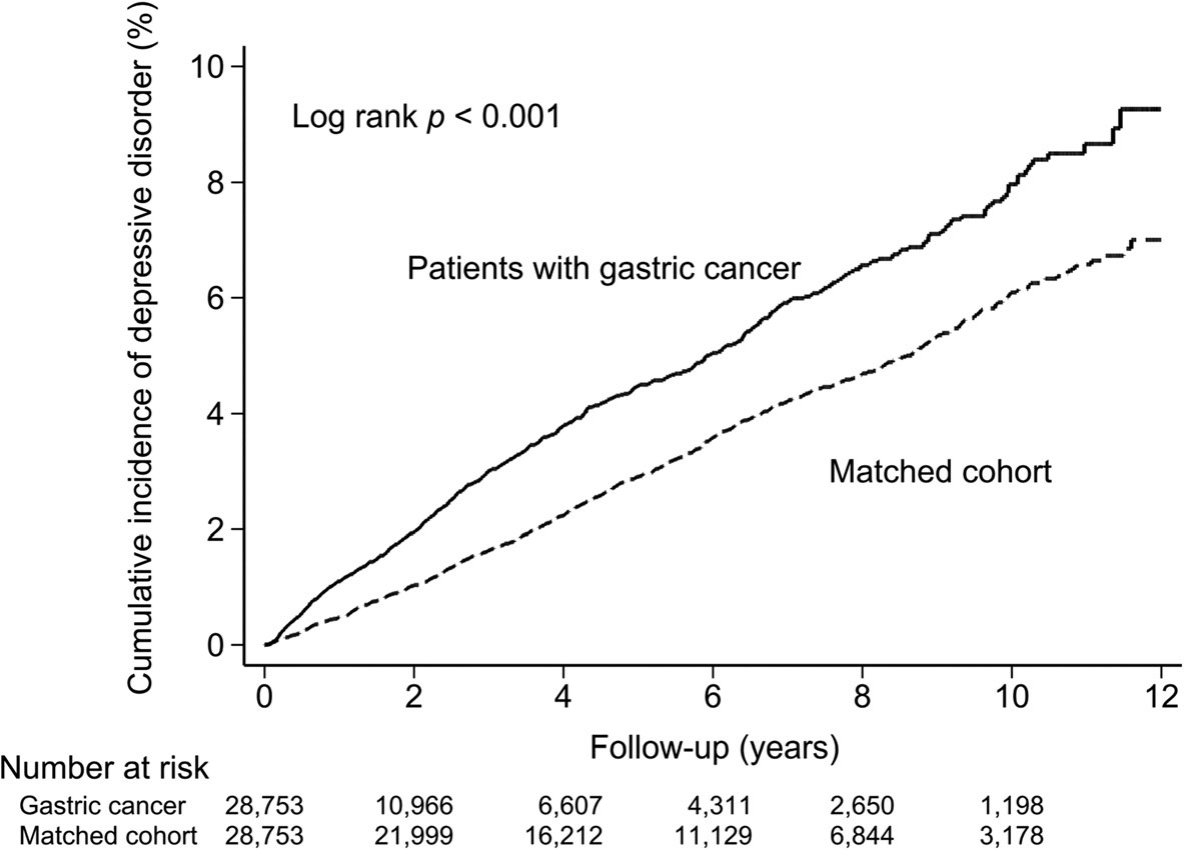
Cumulative incidence of depressive disorders among patients with gastric cancer and matched cohort

Of the 57,506 patients, 1545 patients (2.6%) were diagnosed with depressive disorders; 670 patients with depressive disorders belonged to the gastric cancer cohort (9.1 per 1000 person-years), and 875 belonged to the matched cohort (6.0 per 1000 person-years, *P* < .001). The results of the cumulative incidence of depressive disorders in the gastric cancer cohort and matched cohort are shown in Fig. 1. The cumulative incidence of depressive disorders was significantly higher in the gastric cancer patients compared to those in the matched cohort (*P* < .001, Log-rank test). In addition, the results of the Fine-Gray competing events model revealed that the observed risk of the depressive disorders among gastric cancer patients was still significantly higher than the matched cohort (*P* < .001, Gray’s test).

The adjusted hazard ratio (HR) was 1.54 (95% confidence interval, CI = 1.39–1.70, *P* < .001) in the gastric cancer cohort compared with the matched cohort. As shown in Table 2, differences in the HR for depressive disorders were observed when the patients in both cohorts were stratified according to age and sex. The results showed that younger patients (age < 65 years old) (HR 2.05, 95% CI = 1.75–2.41, *P* < .001) had a higher HR for depressive disorders compared to those who were older (HR 1.27, 95% CI = 1.11–1.45, *P* < .001). When patients were stratified according to sex, female patients (HR 1.80, 95% CI = 1.53–2.12, *P* < .001) had a higher HR compared to the male patients (HR 1.40, 95% CI =1.23–1.59, *P* < .001).

**Table 2:** Incidence of depressive disorders occurrence in patients with and without gastric cancer

	Patients with gastric cancer		Matched cohort		Crude HR (95% CI)	*P* value	Adjusted HR^a^ (95% CI)	*P* value
	Event No.	Per 1000 person-year	Event No.	Per 1000 person-year				
Depressive disorder								
Total	670	9.1	875	6.0	1.52 (1.38–1.69)	< 0.001	1.54 (1.39–1.70)	< 0.001
Age								
≥65	348	9.4	584	7.4	1.26 (1.10–1.44)	< 0.001	1.27 (1.11–1.45)	< 0.001
< 65	322	8.8	291	4.3	2.81 (1.76–2.43)	< 0.001	2.05 (1.75–2.41)	< 0.001
Sex								
Male	389	8.0	565	5.8	1.37 (1.21–1.56)	< 0.001	1.40 (1.23–1.59)	< 0.001
Female	281	11.2	310	6.2	1.79 (1.52–2.11)	< 0.001	1.80 (1.53–2.12)	< 0.001

*HR* incidence hazard ratio, *CI* indicates confidence interval

^a^Adjusted for age, sex, diabetes mellitus, hypertension, heart failure, chronic obstructive pulmonary disease, chronic kidney disease, cirrhosis, autoimmune diseases, cerebrovascular disease and ischemic heart disease

### Risk factors for depressive disorders in the gastric cancer cohort

In Table 3, we applied univariate analysis when trying to predict the development of depressive disorders in the gastric cancer cohort. The results indicated that female sex and some comorbidities—including diabetes mellitus, hypertension, heart failure, COPD, chronic kidney disease, cirrhosis, cerebrovascular disease, and ischemic heart disease—were possible risk factors (*P* < .1).The multivariate analysis confirmed that the following were independent risk factors for depressive disorders among patients with gastric cancer: female sex (HR = 1.46, 95% CI = 1.25–1.70, *P* < .001) and hypertension (HR = 1.27, 95% CI = 1.07–1.52, *p* = .008).

**Table 3:** Analyses of risk factors for depressive disorder in patients with gastric cancer

Predictive variables	Univariate analysis		Multivariate analysis^a^	
	HR (95% CI)	*P* value	HR (95% CI)	*P* value
Age ≥ 65	1.04 (0.89–1.21)	0.611		
Sex (female)	1.41 (1.21–1.64)	< 0.001	1.46 (1.25–1.70)	< 0.001
Comorbidities				
Diabetes mellitus	1.33 (1.12–1.57)	0.001	1.13 (0.94–1.35)	0.186
Hypertension	1.45 (1.24–1.68)	< 0.001	1.27 (1.07–1.52)	0.008
Heart failure	1.41 (1.12–1.77)	0.003	1.12 (0.87–1.43)	0.393
COPD	1.22 (1.03–1.44)	0.019	1.11 (0.93–1.32)	0.259
Chronic kidney disease	1.21 (0.97–1.51)	0.098	1.02 (0.81–1.28)	0.886
Cirrhosis	1.40 (0.99–1.98)	0.056	1.35 (0.95–1.91)	0.090
Autoimmune diseases	1.25 (0.92–1.68)	0.150		
Cerebrovascular disease	1.44 (1.19–1.75)	< 0.001	1.22 (1.00–1.50)	0.053
Ischemic heart disease	1.30 (1.10–1.53)	0.002	1.06 (0.88–1.28)	0.558
Treatment^b^				
Surgery alone	1.03 (0.89–1.21)	0.673		
Surgery with neoadjuvant and/or adjuvant chemoradiotherapy	1.11 (0.96–1.30)	0.167		
Chemoradiotherapy without surgery	0.79 (0.57–1.10)	0.164		

*COPD* chronic obstructive pulmonary disease

^a^All factors with *P* < 0.1 in univariate analyses were included in the Cox multivariate analysis

^b^Treatment was analyzed as a time-dependent covariate in the Cox regression model

## Discussion

The major finding of our study was that the risk of clinical depressive disorders requiring psychiatric intervention was higher among patients with gastric cancer compared to the matched cohort. In addition, female sex and hypertension were the risk factors for the development of subsequent depressive disorders following diagnoses of gastric cancer.

The current study design involved an unbiased participant selection process and used an age-, sex-, and comorbidity-matched cohort as the control group. Because participation in the NHI is mandatory, and nearly all Taiwanese residents can access healthcare with low copayments, referral bias is not a concern and follow-up compliance is high. Furthermore, to apply for a cancer catastrophic illness certificate, pathologic proof of malignancy is mandatory, and laboratory and imaging studies must be provided. Therefore, cancer diagnoses in this study were reliable. The strengths of this study were its large sample size, the long-term (12-year) follow-up period, and clinical depressive disorder diagnoses as defined based on the ICD-9-CM codes and antidepressant prescriptions.

Consistent with the results of previous studies, we observed that the risk of depressive disorders in gastric cancer patients was higher than it was in the control patients. The result may be explained by several possible factors. First, gastric cancer is often diagnosed at a late stage and its outcome is often poor with a 5-year survival rate under 10% [21]; therefore, patients with gastric cancer may have a higher risk of experiencing emotional distress. Second, while the type of surgical procedure does not appear to impact quality of life (QOL), different types of gastric resection may affect eating behavior and physical and emotional functioning [22]. For example, dumping syndrome is more common with a distal gastrectomy, and reflux is more common if the gastroesophageal junction is included in the specimen. These problems may lead to depressive disorders and other QOL problems. Third, studies have shown that patients with gastric cancer exhibit higher levels of mixed depression and anxiety symptoms compared to those with other types of cancer, raising the question of whether gastric cancer is associated with more biological mechanisms of depression, such as cytokine release [23]. Consistent with this biological hypothesis, Koh et al. investigated the association between the brain-derived neurotrophic factor (BDNF) Val66Met polymorphism and stress coping response in patients diagnosed with gastric cancer and found that the BDNF Val66Met polymorphism may be involved in individual coping responses to cancer [24].

Previous studies have reported that depressive symptoms were frequently noted in gastric cancer patients [23, 25]. Although our results are consistent with such findings, the estimated incidence rate of depression among gastric cancer patients in these studies was approximately 20% [25]; this rate is much higher than the rate calculated in our study. There are two possible reasons for the discrepancy between these findings. First, in previous studies, rating scales—such as the HAMD-24 questionnaire—rather than clinical diagnostic interviews, were used to identify depression. Patients who were recorded as depressed may not have fulfilled the diagnostic criteria for depressive disorders from a psychiatric perspective. In our study, we focused on the patients with depressive disorders who were prescribed antidepressants for at least 30 days by clinical oncologists or psychiatrists. Therefore, our study may reflect the real conditions of the gastric cancer patients under the care of clinical conditions. Second, cultural differences may impact the reporting of depressive symptoms in cancer patients [26, 27]. Studies have shown that Asian patients focus more on their physical rather than their psychological symptoms [28]. Moreover, in addition to the difficulty evaluating the etiology of physical symptoms in diagnosing depressive disorders, oncology specialists may also be uncertain regarding the effectiveness of treatment for depressive disorder, limiting referrals for psychiatric interventions. Therefore, gastric cancer patients with depressive disorders may have been underestimated in our study.

In our analysis of the risk factors associated with subsequent depressive disorders among patients with gastric cancer, female sex and hypertension were independent risk factors. The prevalence of hypertension was about 27.2% in 2000 in Asia [29], and this has been increasing annually [30]. From a psychosomatic perspective, hypertension could be concomitant with depression [31]. Depression is closely associated with hypertension. Depression can promote the occurrence and development of hypertension [32]. Meanwhile, hypertension is prone to aggravate depression, and the increased prevalence of depression has been described among patients with hypertension. Rabkin et al. found a three-fold higher frequency of major depressive disorder in patients treated for hypertension [33]. These findings are in line with our study results, which indicated that hypertension may be a risk factor for subsequent depression among patients with gastric cancer. In addition, the female sex was at a greater risk for developing depressive disorders in the present study. This result is in line with previous studies in which the female sex was a risk factor for depression in patients with cancer [34]. Moreover, the results of the analysis of the risk factors showed that treatment regimens were not associated with the increased risk of depressive disorders among patients with gastric cancer; these results seem consistent with earlier research on esophageal cancer patients [16]. However, these results also highlight the complex life-life clinical situation and we should interpret the results carefully. For example, a gastric cancer patient who is eligible to receive curative surgery may have increased feelings of hope, possibly associated with the knowledge that the curative treatment implies a potential chance of a cure; however, the upcoming surgery and poor prognosis, despite undergoing curative treatment, may be reflected in heightened emotional distress. In addition, for those with gastric cancer that is diagnosed late and where surgical extirpation is unlikely, patients may focus on their physical illness needs and be reluctant or even hostile when asked about their psychological needs. These patients with later-stage gastric cancer were also expected to have a shorter life expectancy and, therefore, had fewer chances to receive psychiatric intervention.

Our study is one of the few nationwide studies to examine the association between gastric cancer and subsequent depressive disorders. Its strength is that it used a retrospective matched-cohort study design with a nationwide cohort of patients with gastric cancer and adequate controls for comorbidities. However, several limitations inherent to the use of medical care claims databases should be considered. First, depressive disorder causes are generally complex and vary depending on the patient. Many psychological and environmental factors can contribute to the development of these disorders. We acknowledge that several essential demographic variables—such as a family history of psychiatric disorders, stressful life situations, interpersonal relationships, and lifestyle—were unavailable in the medical care claims database. Second, the cancer stage could not be ascertained and, therefore, whether the severity of the gastric cancer influences the risk of developing depressive disorders warrants further study. Third, as mentioned previously, oncology specialists may view the depressive symptoms of gastric cancer patients as physical discomfort caused by gastric cancer and thus overlook these symptoms. The incidence of depressive disorders in our study may have been underestimated.

## Conclusions

The study suggests that patients with gastric cancer may have more risk of developing subsequent depressive disorders compared with matched cohort. Although it was difficult to provide a clear mechanism to explain the possible role of female sex and hypertension on the association between the gastric cancer and subsequent depression, based on our data, we still suggest that additional attention should focus on both of them. Additional prospective clinical studies of the association between gastric cancer and depressive disorders are needed.

## Abbreviations

aHR: Adjusted hazard ratio
BDNF: Brain-derived neurotrophic factor
CI: Confidence interval
COPD: Chronic obstructive pulmonary disease
HR: Hazard ratio
ICD-9-CM: The International Classification of Diseases, ninth revision, Clinical Modification
NHI: National Health Insurance
NHIRD: National Health Insurance Research Database
